# Spinal Anesthesia and Digital Anxiolysis (SPIDA) for the treatment of lumbar spinal stenosis – a feasibility study

**DOI:** 10.1007/s10143-025-03433-0

**Published:** 2025-03-22

**Authors:** Anton Früh, Andreas Wetzel-Yalelis, Claudius Jelgersma, David Wasilewski, Clara F. Weber, Peter Truckenmueller, Joan Alsolivany, Christian Uhl, Kiarash Ferdowssian, Robert Mertens, Ahmad Almahozi, Jan Arne Blanke, Anika Müller, Alawi Lütz, Nils Hecht, Peter Vajkoczy, Lars Wessels

**Affiliations:** 1 Department of Neurosurgery, Charité - Universitätsmedizin Berlin, Berlin, Germany; 2 Department of Anesthesiology and Operative Intensive Care Medicine, Charité - Universitätsmedizin Berlin, Berlin, Germany

**Keywords:** Lumbar spinal stenosis, Microsurgical decompression, Spinal anesthesia, Awake, Digital anxiolysis

## Abstract

Lumbar spinal stenosis (LSS) is a prevalent condition, particularly in elderly patients, characterized by a clinical syndrome that includes buttock or lower extremity pain, commonly associated with sensory and motor dysfunction. The surgical management of these patients is challenging due to higher rates of comorbidities and increased risks of experiencing complications such as postoperative delirium, leading to intensive care and prolonged hospital stays. Awake surgery under spinal anaesthesia (SA) has been associated with advantages concerning the occurrence of complications and the clinical outcome after surgery. Within this study, we aim to investigate the combination of spinal anesthesia without administration of any systematically effective medication and digital anxiolysis for patients suffering from one-level lumbar spinal stenosis who were treated via microsurgical decompression. This is a single-centre feasibility study. We included patients with LSS that were treated via microsurgical decompression. The patients were divided into groups according to the anaesthetic technique employed: (a) classical GA-Group or (b) Spinal Anaesthesia and Digital Anxiolysis via Virtual Reality Goggles (SPIDA-Group). Demographic, clinical, and radiographic patient data were retrospectively extracted from clinical records and documentation. For SPIDA-Group patients Odom’s criteria (excellent/good/fair/poor) were additionally routinely assessed. Matched pair analysis was performed to compare the outcomes of both groups. The final study population consisted of 65 patients. The surgical and clinical outcomes between GA-Group and SPIDA-Group were equivalent. 86.7% of the SPIDA-Group patients described their surgical experience as excellent, and 13.3% described it as good. All patients treated with the SPIDA-Bundle indicated that they would undergo the surgery again in the SPIDA setting. In 2 patients, the lumbar puncture was unsuccessful (punctio sicca), leading to the switch to GA. One patient reported an inadequate sensitive distribution of the spinal anesthetic, necessitating a switch to GA as well. Furthermore, in one patient, the intrathecal application caused a dural leak. This had to be surgically closed with sutures during the surgery. None of the patients suffered postoperative delirium. The combination of digital anxiolysis and spinal anesthesia is a feasible and promising approach for the microsurgical treatment of LSS. Patients report compelling satisfaction, and clinical outcomes are comparable to GA.

## Introduction

Lumbar spinal stenosis (LSS) is a prevalent condition, particularly in elderly patients, characterized by a clinical syndrome that includes buttock or lower extremity pain, commonly associated with sensory and motor dysfunction [1, 2]. The hallmark of this condition is neurogenic claudication, marked by exacerbated symptoms during ambulation [2, 3]. The prevalence is estimated to be approximately 11% in US adults [4]. Notably, LSS predominantly affects elderly patients, emerging as the primary cause of spinal surgery in patients older than 65 [5].

Given the limited efficacy of conservative treatments, microsurgical decompression has emerged as a preferred therapeutic strategy with good clinical results [6]. Especially the surgical management of elderly patients is challenging due to higher rates of comorbidities and increased risks of experiencing complications such as postoperative delirium, leading to intensive care and prolonged hospital stays [7]. This highlights the necessity for continued research and development in surgical practices and patient care protocols to mitigate such adverse outcomes. Despite advancements in microsurgical techniques, the patient experience during and after the procedure remains an area for potential improvement. Awake surgery under spinal anaesthesia has been associated with advantages concerning the occurrence of complications and the clinical outcome after surgery [7–10]. A Meta-Analysis showed that patients undergoing awake surgery under spinal anaesthesia had shorter duration of surgeries, length of hospital stays and fewer rates of postoperative nausea compared to subjects treated with general anesthesia [11]. However, existing studies show variations regarding the use of additional systematic medication for sedation and anxiolysis, that potentially are drivers of postoperative delirium and cognitive impairments [12–15]. Especially in elderly patients the incidence of preexisting and secondary diseases is high, leading to an elevated risk profile regarding complications [16, 17]. Further, alleviating the effect on pain and anxiety during needle-related procedural pain has been described for the use of Virtual Reality (VR) [18].

Within this study, we aim to investigate the combination of spinal anesthesia without administration of any systematically effective medication and digital anxiolysis for patients suffering from one-level lumbar spinal stenosis who were treated via microsurgical decompression.

## Methods

### Study design

This is a retrospective single-center feasibility study. The trial was approved by the local ethics committee (EA2/58/24). All patients over the age of 18 years were eligible. All included patients reported classic symptoms of lumbar spinal stenosis. The treating physician made the indication for microsurgical decompression. Since May 2023, our tertiary center has offered awake spinal anesthesia (SA) as an alternative to general anesthesia (GA) for spinal surgery. Data acquisition and presentation were done according to the STROBE criteria [19].

### Interventions

**Fig. 1:**
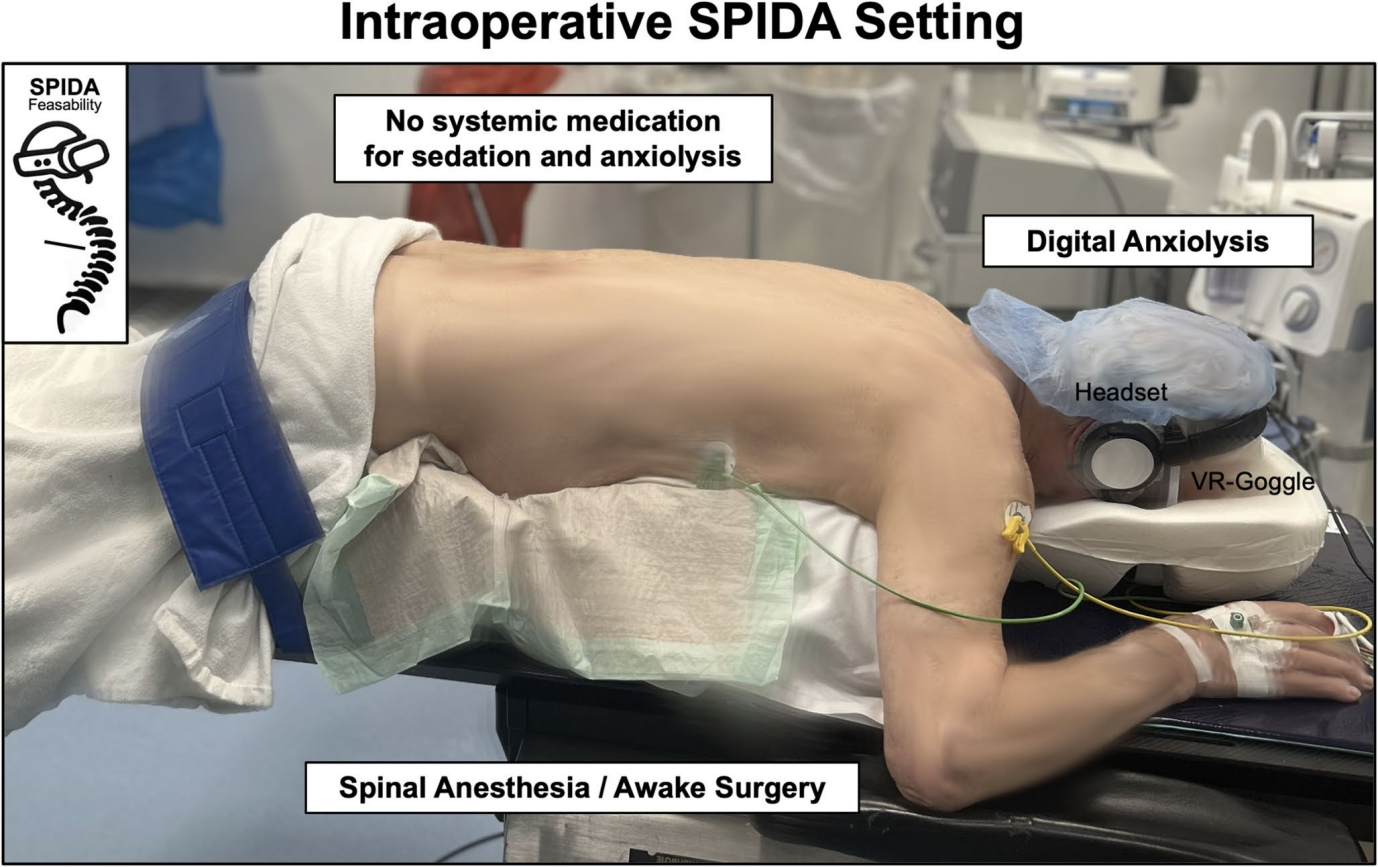
SPIDA-Bundle – Intraoperative setting

VR goggles were used in awake spine surgery patients to create a pleasant, immersive environment. Integrating SA and VR Goggles was defined as “Spinal Anaesthesia and Digital Anxiolysis (SPIDA) - Bundle.” This was intended to distract patients from the stress factors of the surgery and thus improve the subjective experience regarding the procedure. All patients were stratified into two groups based on the anaesthetic technique employed: (a) classical GA (GA-Group) or (b) SPIDA-Bundle (SPIDA-Group). The SPIDA group patients received a single intrathecal dose of spinal anesthesia. This was achieved through the intrathecal administration of isobaric bupivacaine hydrochloride 0.5%, with the dosage determined at the discretion of the attending anesthesiologist in the sitting position. No additional anesthetic agents were used, and no premedication for pharmacological anxiolysis was administered. A MetaQuest 3 (Meta, United States of America) was used as VR headset system. The intraoperative setting of the patients treated with the SPIDA-Bundle is provided in Fig. 1. The patients in this feasibility study had the option to choose between two VR-programs during the procedure. All patients (both groups) received a microsurgical decompression of the spinal canal with state-of-the-art techniques. The selection of the specific surgical approach, selected from options including interlaminar fenestration, hemilaminectomy, and laminectomy, was left to the discretion of the treating surgeon and was independent of the anaesthesia method used.

### Patient cohort and outcomes

We identified all subsequent patients who underwent spinal surgery with the SPIDA-Bundle at our Department of Neurosurgery in Berlin. As a control group (GA-Group), we analyzed 50 patients with GA treated in the same period. Demographic, clinical, and radiographic patient data were retrospectively extracted from clinical records and documentation. In Patients receiving the SPIDA-Bundle surgery, Odom’s criteria (excellent/good/fair/poor) were routinely assessed at discharge. Furthermore, postoperatively, these patients were asked if they would opt for the same surgical procedure again.

### Statistics

Statistical analysis was performed with SPSS version 25 (IBM Corp), R (version 4.3.1), Microsoft Excel 2021, and GraphPad Prism (version 10). Discrete data were presented as counts and percentages and analyzed by using the chi-square test. Continuous data were presented as the median and interquartile range (IQR) and compared using Mann-Whitney, Fisher’s, and Wilcoxon statistics. Two-sided p-values < 0.05 were taken to indicate statistical significance. Matched-Paired analysis was performed based on the patients’ age and ASA-Score with the R-package “MatchIt.”

## Results

**Fig. 2:**
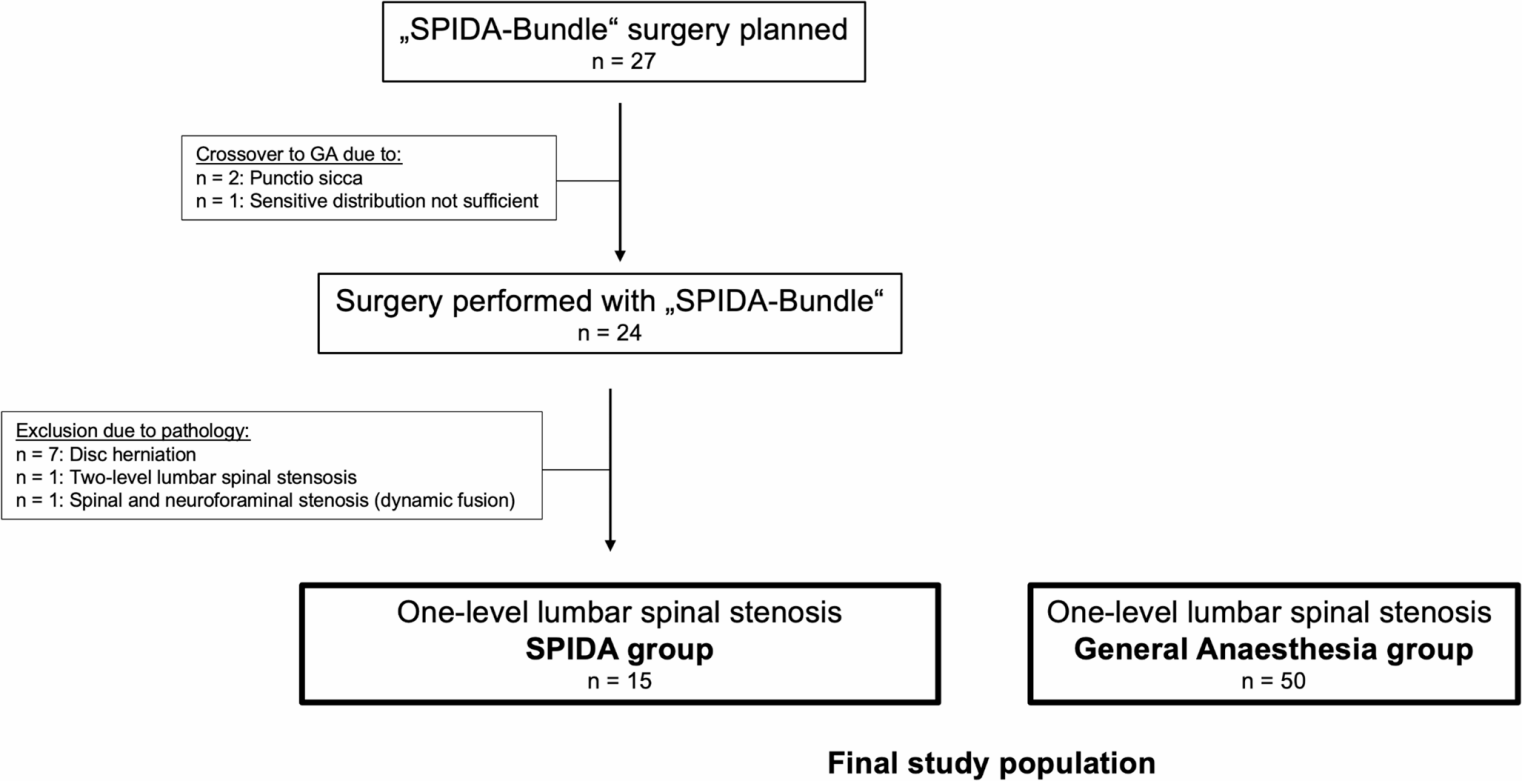
Study flow diagram. n = number

The final study population consisted of 65 patients. In 27 patients, a spinal surgery with the SPIDA-Bundle was planned. Overall, 15 patients received a one-level microsurgical decompression due to an LSS with the SPIDA-Bundle. A flow diagram of the study is illustrated in Fig. 2. The median age of the study population was 73 [IQR 64–83] years, and 31 (47.7%) of the patients were female. The baseline characteristics stratified according to the classification into the SPIDA or GA groups are provided in Table 1.

**Table 1:** Baseline characteristics of the final study population stratified regarding the type of anaesthesia (GA-Group vs. SPIDA-Group)

Characteristic	GA (*n* = 50)	SPIDA (*n* = 15)	*p*-value^T^
Age, Median (IQR)	72 (60–78)	82 (72–86)	0.0023
Sex, n (%)			0.9277
Female	24 (48.0)	7 (46.7)	
Male	26 (52.0)	8 (53.3)	
Location of decompression, n (%)			0.2199
L1/2	1 (2)	0 (0)	
L2/3	4 (8)	1 (7)	
L3/4	26 (52)	4 (27	
L4/5	19 (38)	10 (67)	
Preoperative ASA Score, n (%)			0.8439
1	2 (4)	0 (0)	
2	30 (60)	8 (53)	
3	16 (32)	7 (47)	
4	2 (4)	0 (0)	
aHTN, n (%)	31 (62)	9 (60)	0.8889
DM, n (%)	3 (6)	5 (33)	0.0129
History of smoking, n (%)	7 (14)	1 (7)	0.6694

^T^Welch Two Sample t-test; Pearson’s Chi-squared test; Fisher’s exact test. Abbreviations: GA = General Anaesthesia, n = number, SPIDA = Spinal Anesthesia and Digital Anxiolysis

All patients were treated via interlaminar fenestration (eILF). There is no mortality to report. The results demonstrate high patient satisfaction with the SPIDA-Bundle procedure. Thirteen (86.7%) patients described their surgical experience as excellent, and two (13.3%) described it as good according to the Odom criteria. None of the patients reported experiencing pain during the surgery. No patient wanted to refuse spinal anesthesia during the operation. All 15 patients treated with the SPIDA-Bundle indicated that they would undergo the surgery again in the SPIDA setting. The surgical and clinical outcomes between SPIDA-Group and GA-Group are equivalent. There was no difference in the duration of the surgery or the duration of the treatment by the anesthesiologist. None of the patients suffered from a postoperative delirium, and the length of hospital stay was also the same for both groups. The outcome of the whole study population is demonstrated in Table 2.

**Table 2:** Outcome of the final study population stratified regarding the type of anaesthesia (GA vs. SPIDA)

Characteristic	GA (*n* = 50)	SPIDA (*n* = 15)	*p*-value^T^
Duration of surgery (min), Median (IQR)	83 (53–116)	82 (47–97)	0.29
Time of patient in the operating theater, Median (IQR)	127 (105–170)	120 (100–140)	0.41
Duration of treatment by the anesthesiologist, Median (IQR)	153 (123–199)	153 (132–164)	0.53
Unknown	0	1	
Dura leakage due to surgery, n (%)	8 (16)	1 (6.7)	0.67
Surgery associated complication, n (%)	1 (2.0)	1 (6.7)	0.41
Postoperative Delir, n (%)	0 (0)	0 (0)	
Reoperation during stay, n (%)	0 (0)	1 (6.7)	0.23
Length of stay, Median (IQR)	3 (2–4)	2 (2–5)	0.50
Type of discharge, n (%)			0.23
Home	50 (100)	14 (93)	
Hospital	0 (0)	1 (6.7)	

^T^Welch Two Sample t-test; Pearson’s Chi-squared test; Fisher’s exact test. Abbreviations: GA = General Anaesthesia, IQR = Interquartile Range, min = minute, n = number, SPIDA = Spinal Anesthesia and Digital Anxiolysis

### Special consideration and complications associated with the SPIDA-bundle

The comprehensive analysis of all patients scheduled for surgery with the SPIDA-Bundle (*n* = 27) at our tertiary center revealed that three patients required a crossover to GA. In 2 patients, the lumbar puncture was unsuccessful (punctio sicca), leading to the switch to GA. One patient reported an inadequate sensitive distribution of the spinal anesthetic, necessitating a switch to GA as well. Furthermore, in one patient, the intrathecal application caused a dural leak. This had to be surgically closed with sutures during the surgery. The patient exhibited no postoperative morbidity. Three of the 24 patients (12.5%) who operated with the SPIDA-Bundle indicated they did not want to wear the VR goggles to be more aware of the surgical procedure. In these cases, the use of VR goggles during the operation was omitted.

### Matched paired analysis

As the mean age significantly differed between the groups, an additional matched-pair analysis was performed based on the age and the ASA classification of the patients. The corresponding baseline characteristics of the matched patients are provided in Table 3 and show no differences. These patients’ outcomes remain equal between both groups (see Table 4).

**Table 3:** Baseline characteristics of matched patients (age and ASA-Score) stratified regarding the type of anaesthesia (GA vs. SPIDA)

Characteristic	GA matched paired (*n* = 15)	SPIDA (*n* = 15)	*p*-value^T^
Age, Median (IQR)	82 (72–83)	82 (72–86)	0.786
Sex, n (%)			1.000
Female	7 (46.7)	7 (46.7)	
Male	8 (53.3)	8 (53.3)	
Location of decompression, n (%)			0.2199
L1/2	1 (7)	0 (0)	
L2/3	1 (7)	1 (7)	
L3/4	8 (53)	4 (27)	
L4/5	5 (33)	10 (67)	
Preoperative ASA Score, n (%)			1.000
2	8 (53)	8 (53)	
3	7 (47)	7 (47)	
aHTN, n (%)	11 (73)	9 (60)	0.439
DM, n (%)	0 (0)	5 (33)	0.042
History of smoking, n (%)	0 (0)	1 (7)	1.000

^T^Welch Two Sample t-test; Pearson’s Chi-squared test; Fisher’s exact test. Abbreviations: GA = General Anaesthesia, n = number, SPIDA = Spinal Anesthesia and Digital Anxiolysis

**Table 4:** Outcome of the matched patients stratified regarding the type of anaesthesia (GA vs. SPIDA)

	GA matched paired (*n* = 15)	SPIDA (*n* = 15)	*p*-value^T^
Duration of surgery (min), Median (IQR)	65 (50–102)	82 (47–97)	0.88
Time of patient in the operating theater, Median (IQR)	112 (97–143)	120 (100–140)	0.77
Duration of treatment by the anesthesiologist, Median (IQR)	141 (113–168)	153 (132–164)	0.79
Unknown	0	1	
Dura leakage due to surgery, n (%)	3 (20)	1 (6.7)	0.60
Surgery associated complication, n (%)	0 (0)	1 (6.7)	1.00
Postoperative Delir, n (%)	0 (0)	0 (0)	
Reoperation during stay, n (%)	0 (0)	1 (6.7)	1.00
Length of stay, Median (IQR)	4 (3–4)	2 (2–5)	0.11
Type of discharge, n (%)			1.00
Home	15 (100)	14 (93)	
Hospital	0 (0)	1 (6.7)	

^T^Welch Two Sample t-test; Pearson’s Chi-squared test; Fisher’s exact test. Abbreviations: GA = General Anaesthesia, IQR = Interquartile Range, min = minute, n = number, SPIDA = Spinal Anesthesia and Digital Anxiolysis

## Discussion

This is the first study showing the feasibility of the combination of digital anxiolysis and spinal anesthesia for the microsurgical treatment of LSS. Patients treated with the SPIDA-Bundle demonstrated compelling satisfaction with the procedure, with further outcome parameters comparable to those with General Anesthesia. Therefore, this approach is worth further prospective, multicenter, and randomized investigations in the future.

LSS is common, especially in elderly patients [1, 2]. Thereby, patients suffering from both severe leg and back pain benefit from surgical decompressions [20]. In neurosurgery, particularly lumbar operations, the evolution of surgical and anesthetic techniques has led to the exploration of methods to enhance patient comfort and reduce intraoperative stress. Therefore, awake surgery under SA is an alternative to surgery under GA in neurological and spine surgery [21]. This technique allows for the preservation of consciousness and significantly reduces the risks associated with general anesthesia, especially in patients with complex medical conditions. Particularly due to the increasing life expectancy and the consequent globally rising number of elderly patients undergoing spinal surgeries [22], it appears prudent to reduce the burden of anesthesia during the operative procedures. Meta-analysis encompassing various types of spine surgeries (including fusions, decompressions, and kyphoplasties) demonstrated that patients undergoing awake spine surgery with SA experienced notably shorter durations of operations and hospital stays, along with reduced incidences of postoperative nausea and urinary retention, in comparison to those receiving GA [10, 21]. Despite the potential benefits, the practice of awake spine surgery has yet to gain widespread acceptance. A survey examining surgeons’ perspectives uncovered considerable skepticism [10]. One of the present authors’ concerns was that patients after spinal SA cannot be neurologically examined immediately after the surgery, unlike with GA after extubating. However, in the context of this feasibility study, we have no cases to report where the ability to perform examinations delayed surgical follow-up care, such as for a postoperative hemorrhage. Nevertheless, future studies should remain attentive and investigate events such as the described cerebrospinal fluid leak caused by spinal puncture. Notably, the current study was underpowered for the investigation of complications, necessitating further research in this area.

To further mitigate the psychological discomfort and stress factors associated with surgery, the integration of VR technology presents a novel adjunct. Equipping patients with VR headsets during the procedure allows them to be immersed in a controlled, serene environment that distracts from the operational setting. This experience can range from tranquil natural landscapes to guided meditations, effectively diverting the patient’s attention from the surgery. Systematic medication for sedation and anxiolysis are potent drivers of postoperative delirium and cognitive impairments [12, 15]. The use of virtual reality goggles in the study resulted in high satisfaction levels, with none of the patients requiring anxiolytic medication or sedation. Therefore, a positive effect regarding the incidence of postoperative delirium and cognitive impairments could be possible in subsequent studies. In subsequent research projects, further investigation of the effects of different VR environments on patients would be interesting.

The combination of spinal anesthesia with digital anxiolysis offered by VR technology represents a new approach during the microsurgical treatment of LSS. Overall, the present SPIDA-Bundle demonstrated convincing anxiolytic effects, as the overall satisfaction during the surgery was high. This is in line with a study that reported the positive effects of VR systems for pediatric patients [18]. Some patients declined to wear the VR goggles during surgery to be more aware of the operative proceedings. For these patients, a technical solution could be to offer live images of the operation through the VR goggles. During spinal anesthesia, certain surgical conditions should be carefully considered, as they may significantly prolong the procedure and impact postoperative outcomes. Anatomical variations can present challenges that necessitate intraoperative muscle relaxation, particularly during dissection and exposure of the affected spinal structures. This is especially relevant in obese patients and individuals with well-developed musculature, where achieving adequate surgical exposure may be more difficult. Therefore, meticulous patient selection is essential for the success of awake spinal surgery. Future studies should further explore patient-specific criteria to optimize surgical outcomes and minimize potential complications.

The present study acknowledges certain limitations, primarily its design as a monocentric feasibility study. In the context of this retrospective study, only patients who were willing to receive spinal anesthesia before the operation were treated with the SPIDA-Bundle. Consequently, the satisfaction with the surgery could potentially be overestimated. Additionally, the study was conducted in a tertiary specialized spine center, a setting that may not be representative. Furthermore, spinal anesthesia precludes the use of intraoperative neuromonitoring. However, since neuromonitoring is not routinely performed in the surgical treatment of LSS, this limitation did not impact the patients in the present study. Moreover, due to the retrospective design of the study, precise data on the number of hypotensive episodes during surgery are missing. As intraoperative hemodynamic parameters can affect surgical and cognitive outcomes, this represents a major limitation of the study and will be addressed in future trials. The retrospective design of this study made it challenging to accurately assess patients’ preoperative neurocognitive status. As baseline cognitive function is a key factor in evaluating postoperative cognitive dysfunction and delirium, the absence of a standardized preoperative neurocognitive assessment may have introduced bias in the interpretation of postoperative outcomes. Future prospective studies should incorporate structured cognitive screening tools to better assess the relationship between preoperative cognitive status and postoperative neurological complications. We consider the data from this feasibility study promising, especially given the high patient satisfaction. Therefore, the authors are planning a prospective, randomized, multicenter SPIDA trial.

## Conclusion

The combination of digital anxiolysis and spinal anesthesia is a feasible and promising approach for the microsurgical treatment of LSS. Patients report compelling satisfaction, and clinical outcomes are comparable to GA.

## Data Availability

No datasets were generated or analysed during the current study.
